# One new species of the genus *Belisana* Thorell, 1898 (Araneae, Pholcidae) from northern Vietnam

**DOI:** 10.3897/zookeys.480.9046

**Published:** 2015-02-02

**Authors:** Dinh-Sac Pham

**Affiliations:** 1 Institute of Ecology and Biological Resources, Vietnam Academy of Science and Technology, 18 Hoang Quoc Viet, Cau Giay, Hanoi, Vietnam

**Keywords:** Taxonomy, pholcid, Southeast Asia, fogging, canopy

## Abstract

One new species *Belisana denticulata* **sp. n.** (♂) is reported from northern Vietnam based on material collected by fogging the forest canopy. This species resembles *Belisana scharffi* Huber, 2005, but can be distinguished by relatively long distance between proximal parts of proximo-lateral apophysis and distal apophysis on male chelicerae, by presence of a nearly saddle-shaped prolateral sclerite on procursus, and by different shape of retrolateral membranous flap on procursus. Type specimens are deposited in the Vietnam Academy of Science and Technology in Hanoi.

## Introduction

*Belisana* Thorell, 1898, the second largest genus in Pholcidae C.L. Koch, 1850, includes 110 species (World Spider Catalog 2014, Yao et al. 2015). The genus is highly diverse in Southeast Asia, including 14 species reported from Vietnam. They are: *Belisana babensis* Yao, Pham & Li, 2015, *Belisana cheni* Yao, Pham & Li, 2015, *Belisana clavata* Yao, Pham & Li, 2015, *Belisana curva* Yao, Pham & Li, 2015, *Belisana decora* Yao, Pham & Li, 2015, *Belisana halongensis* Yao, Pham & Li, 2015, *Belisana limpida* (Simon, 1909), *Belisana phungae* Yao, Pham & Li, 2015, *Belisana schwendingeri* Huber, 2005, *Belisana sepaku* Huber, 2005, *Belisana pisinna* Yao, Pham & Li, 2015, *Belisana triangula* Yao, Pham & Li, 2015, *Belisana vietnamensis* Yao, Pham & Li, 2015, *Belisana zhengi* Yao, Pham & Li, 2015.

During examination of the spider material collected by fogging in Tam Dao National Park, a new species of the genus *Belisana* was founded. In the current paper, description of the new species is provided; the detailed structure of pedipalp is photographed and illustrated.

## Material and methods

Specimens were examined and measured with a Leica M205 C stereomicroscope. Details were studied with an Olympus BX51 compound microscope. Illustrations were made using a camera lucida attached to the Olympus BX51 microscope, and inked using an ink jet plotter. Male copulatory organs were examined and illustrated after they were dissected from the spiders. Type specimens were preserved in 75% ethanol solution. Photographs were taken with an Olympus C7070 wide zoom digital camera (7.1 megapixels) mounted on a Leica M205 C stereomicroscope. The images were assembled using Helicon Focus 3.10 image stacking software. All measurements are given in millimeters. Leg measurements are shown as: Total length (femur + patella + tibia + metatarsus + tarsus). Leg segments were measured on their dorsal side. Distribution map was generated with ArcView GIS 3.2. Terminology and taxonomic descriptions follow Huber (2000). Type specimens are deposited in the Institute of Ecology and Biological Resources, Vietnam Academy of Science and Technology in Hanoi, Vietnam.

The following abbreviations are used in the description: ALE = anterior lateral eye, AME = anterior median eye, PME = posterior median eye, L/d = length/diameter.

## Taxonomy

### Genus Belisana

Taxon classificationAnimaliaAraneaePholcidae

Thorell, 1898

#### Type species.

*Belisana tauricornis* Thorell, 1898

### Belisana denticulata sp. n.

Taxon classificationAnimaliaAraneaePholcidae

http://zoobank.org/D82D1866-428A-4F07-B1F9-43E1BD589AC9

Figs 1 , 2 , 3 , 4

#### Type material.

Holotype: ♂, fogging, natural forest (21°31.501'N, 105°33.434'E, elevation 1060 m), Tam Dao National Park, Vinh Phuc, Vietnam, 26 July 2008, D.S. Pham leg. Paratype: 1 ♂, same data but disturbed forest (21°28.337'N, 105°38.094'E, elevation 1007 m).

#### Etymology.

The specific name is derived from the Latin *denticulatus* (with small teeth), in reference to the small teeth on the prolateral sclerite of the distal procursus; adjective.

#### Diagnosis.

**Figure 1.**
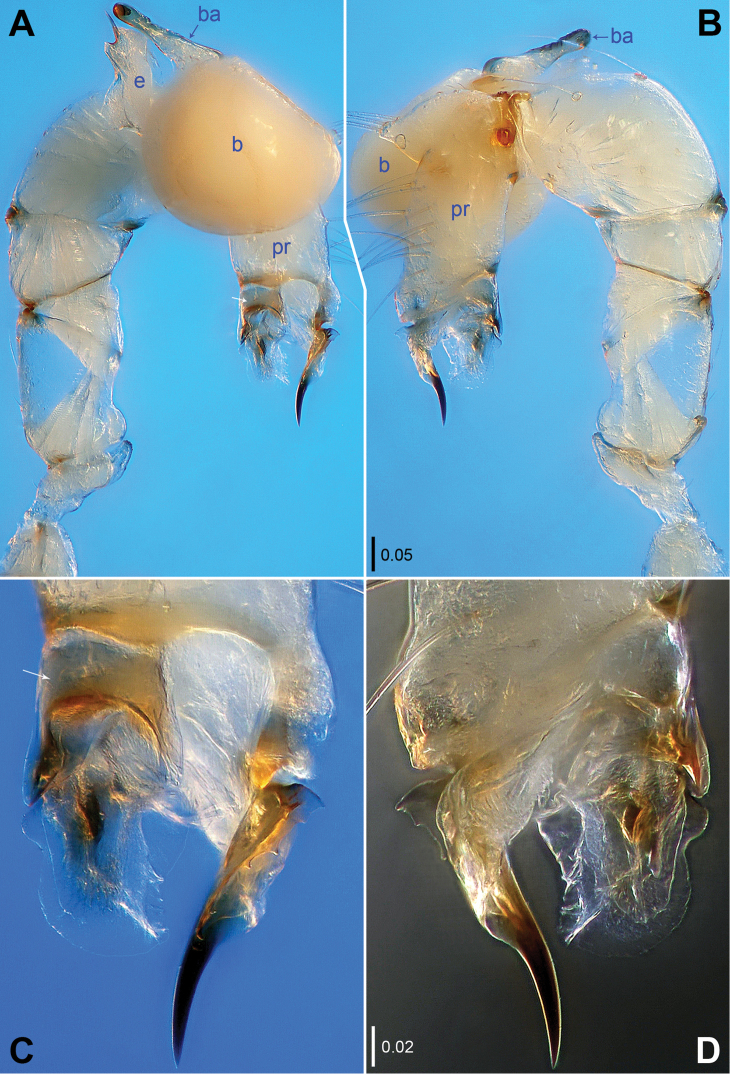
*Belisana denticulata* sp. n., holotype male. **A–B** Pedipalp (**A** Prolateral view, arrow points at nearly saddle-shaped sclerite **B** Retrolateral view) **C–D** Distal part of procursus (**C** Prolateral view, arrow points at nearly saddle-shaped sclerite **D** Retrolateral view). **b** = bulb, **ba** = bulbal apophysis, **e** = embolus, **pr** = procursus.

**Figure 2.**
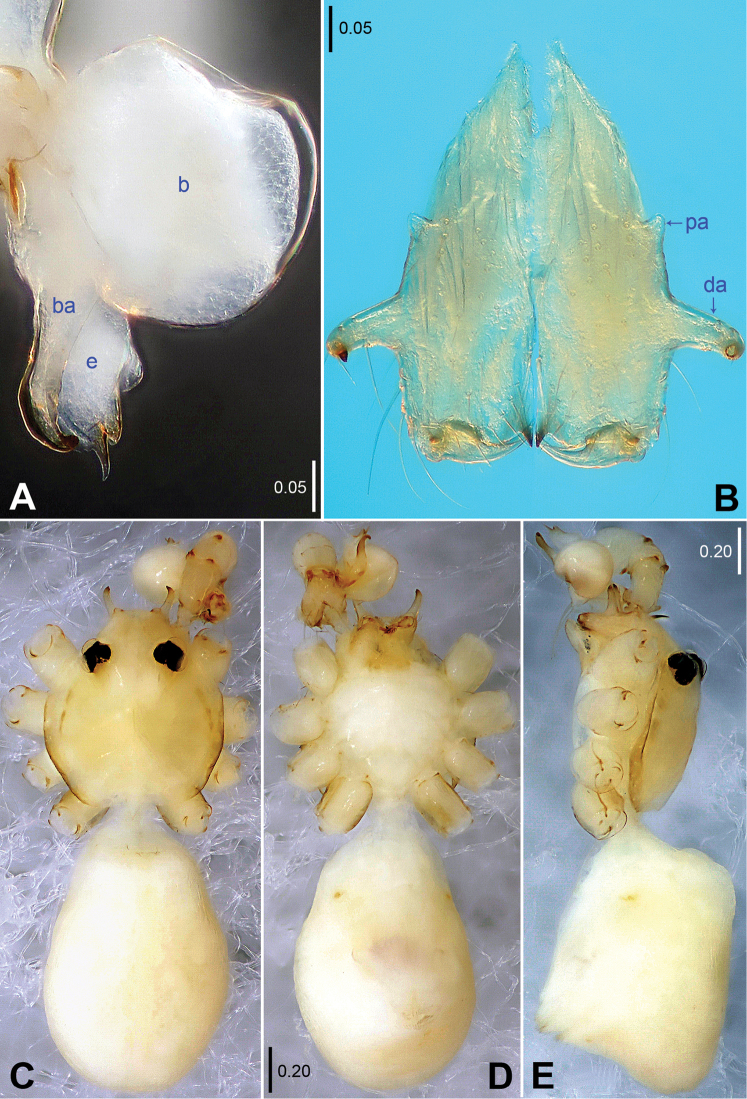
*Belisana denticulata* sp. n., holotype (**B–E**) and paratype (**A**) males. **A** Bulb, prolateral view **B** Chelicerae, frontal view **C–E** Habitus (**C** Dorsal view **D** Ventral view **E** Lateral view). **b** = bulb, **ba** = bulbal apophysis, **da** = distal apophysis, **e** = embolus, **pa** = proximo-lateral apophysis.

**Figure 3.**
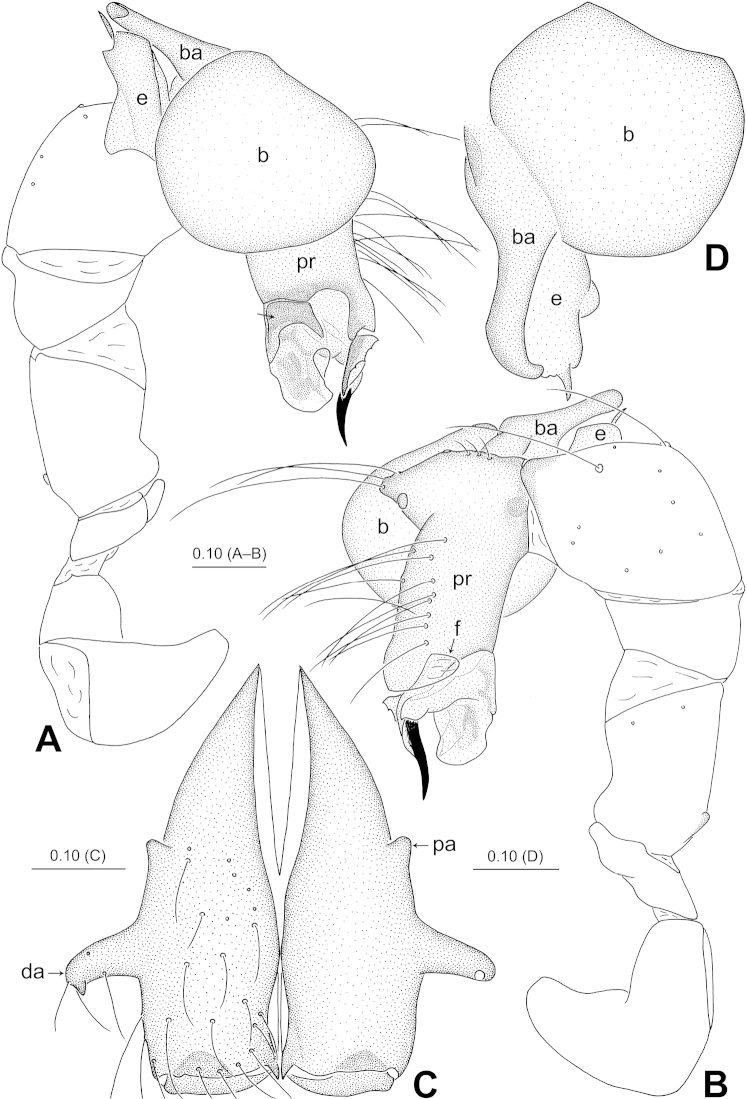
*Belisana denticulata* sp. n., holotype (**A–C**) and paratype (**D**) males. **A–B** Pedipalp (**A** Prolateral view, arrow points at nearly saddle-shaped sclerite **B** Retrolateral view) **C** Chelicerae, frontal view **D** Bulb, prolateral view. **b** = bulb, **ba** = bulbal apophysis, **e** = embolus, **f** = membranous flap, **pr** = procursus.

This species resembles *Belisana scharffi* Huber, 2005 (see Huber 2005: 20, figs 9–10, 65, 136–153), but can be distinguished by relatively long distance between proximal parts of proximo-lateral apophysis and distal apophysis on male chelicerae (Figs 2B, 3C), by presence of a nearly saddle-shaped prolateral sclerite on procursus (arrows in Figs 1A, C, 3A), and by different shape of retrolateral membranous flap on procursus (Fig. 3B).

#### Description.

**Male (holotype):** Total length 1.97 (2.09 with clypeus), prosoma 0.74 long, 0.80 wide, opisthosoma 1.23 long, 0.84 wide. Leg I: – (3.72 + 0.36 + – + – + –), leg II: 10.78 (2.95 + 0.34 + 2.62 + 3.89 + 0.98), leg III: 6.96 (1.87 + 0.30 + 1.72 + 2.41 + 0.66), leg IV: 9.22 (2.60 + 0.31 + 2.34 + 3.25 + 0.72). Habitus as in Fig. 2C–E. Dorsal shield of prosoma yellowish, with brown lateral margins; sternum yellowish, without marks. Legs yellowish, without darker rings. Opisthosoma yellowish, without spots. Distance PME-PME 0.21, diameter PME 0.09, distance PME-ALE 0.02, AME absent. Ocular area not elevated. Thoracic furrow absent. Clypeus unmodified. Sternum wider than long (0.61/0.57). Chelicerae (Figs 2B, 3C) with a pair of thumb-shaped proximo-lateral apophyses and a pair of long, curved distal apophyses (distance between tips: 0.42). Pedipalps as in Figs 1A–B, 3A–B; trochanter with a short retrolatero-ventral apophysis; femur with a small dorsal apophysis; procursus simple proximally but complex distally, with a prolateral sclerite provided with small teeth, a small membranous flap retrolaterally and a curved spine; bulb with a hooked apophysis and a simple embolus. Legs with short vertical hairs on metatarsi, without spines and curved hairs.

**Variation.** In another male: Tibia I: 3.37; tibia I L/d: 45. Retrolateral trichobothrium of tibia I at 13% proximally; tarsus I with about 12 distinct pseudosegments.

**Female.** Unknown.

#### Distribution.

**Figure 4.**
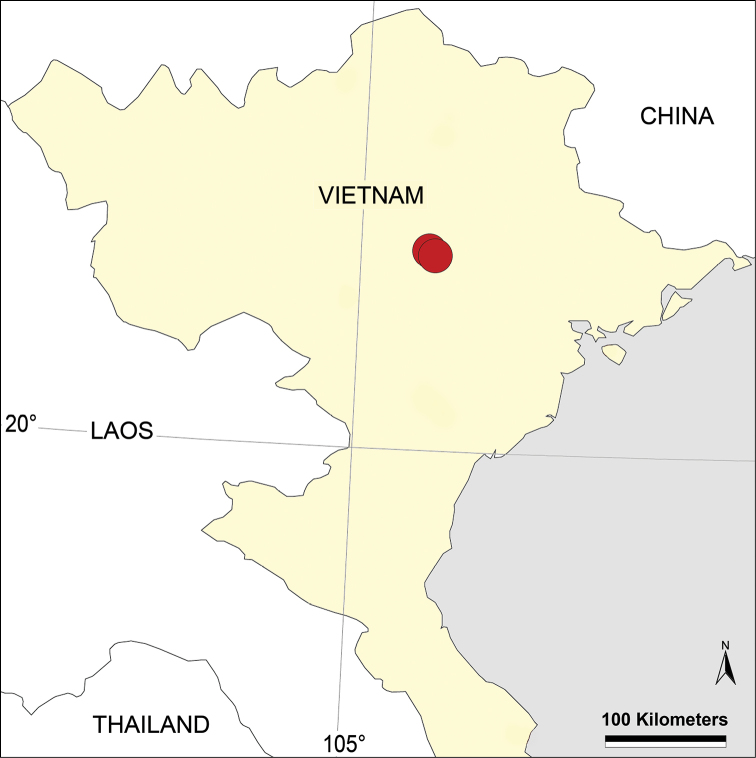
Distribution records of *Belisana denticulata* sp. n. from northern Vietnam.

Known from two nearby localities in Tam Dao National Park (Fig. 4).

## Supplementary Material

XML Treatment for Belisana

XML Treatment for Belisana denticulata

## References

[B1] Huber BA (2000) New World pholcid spiders (Araneae: Pholcidae): A revision at generic level. Bulletin of the American Museum of Natural History 254: 1–348. doi: 10.1206/0003-0090(2000)254<0001:NWPSAP>2.0.CO;2

[B2] Huber BA (2005) High species diversity, male-female coevolution, and metaphyly in Southeast Asian pholcid spiders: the case of *Belisana* Thorell 1898 (Araneae, Pholcidae). Zoologica 155: 1–126.

[B3] World Spider Catalog (2014) World Spider Catalog, version 15.5. Natural History Museum Bern http://wsc.nmbe.ch [accessed 30 November 2014]

[B4] Yao Z Pham DS Li S (2015) Pholcid spiders (Araneae: Pholcidae) from northern Vietnam, with descriptions of nineteen new species. Zootaxa 3909(1): 1–82. doi: 10.11646/zootaxa.3909.1.1 10.11646/zootaxa.3909.1.1 25661430

